# Argon plasma surface modification promotes the therapeutic angiogenesis and tissue formation of tissue-engineered scaffolds in vivo by adipose-derived stem cells

**DOI:** 10.1186/s13287-019-1195-z

**Published:** 2019-03-29

**Authors:** M. F. Griffin, N. Naderi, D. M. Kalaskar, A. M. Seifalian, P. E. Butler

**Affiliations:** 1 UCL Centre for Nanotechnology and Regenerative Medicine, Division of Surgery and Interventional Science, University College London, London, UK; 2 Royal Free London NHS Foundation Trust Hospital, London, UK; 3 Charles Wolfson Center for Reconstructive Surgery, Royal Free Hospital, London, UK; 4 UCL Institute of Orthopaedics and Musculoskeletal Science, Division of Surgery and Interventional Science, University College London, Stanmore, Middlesex, HA7 4LP UK; 5 Nanotechnology and Regenerative Medicine Commercialization Centre (Ltd), The London Bioscience Innovation Centre, London, NW1 0NH UK; 6 Plastic and Reconstructive Surgery Department, Royal Free Hospital, University College London, Pond Street, London, UK

**Keywords:** Adipose-derived stem cell, Angiogenesis, Tissue integration, Vascular-derived growth factor, Plasma surface modification

## Abstract

**Background:**

Synthetic implants are being used to restore injured or damaged tissues following cancer resection and congenital diseases. However, the survival of large tissue implant replacements depends on their ability to support angiogenesis that if limited, causes extrusion and infection of the implant. This study assessed the beneficial effect of platelet-rich plasma (PRP) and adipose-derived stem cells (ADSCs) on synthetic biomaterials in combination with argon plasma surface modification to enhance vascularisation of tissue-engineered constructs.

**Methods:**

Non-biodegradable polyurethane scaffolds were manufactured and modified with plasma surface modification using argon gas (PM). Donor rats were then used to extract ADSCs and PRP to modify the scaffolds further. Scaffolds with and without PM were modified with and without ADSCs and PRP and subcutaneously implanted in the dorsum of rats for 3 months. After 12 weeks, the scaffolds were excised and the degree of tissue integration using H&E staining and Masson’s trichrome staining, angiogenesis by CD31 and immune response by CD45 and CD68 immunohistochemistry staining was examined.

**Results:**

H&E and Masson’s trichrome staining showed PM+PRP+ADSC and PM+ADSC scaffolds had the greatest tissue integration, but there was no significant difference between the two scaffolds (*p* < 0.05). The greatest vessel formation after 3 months was shown with PM+PRP+ADSC and PM+ADSC scaffolds using CD31 staining compared to all other scaffolds (*p* < 0.05). The CD45 and CD68 staining was similar between all scaffolds after 3 months showing the ADSCs or PRP had no effect on the immune response of the scaffolds.

**Conclusions:**

Argon plasma surface modification enhanced the effect of adipose-derived stem cells effect on angiogenesis and tissue integration of polyurethane scaffolds. The combination of ADSCs and argon plasma modification may improve the survival of large tissue implants for regenerative applications.

**Electronic supplementary material:**

The online version of this article (10.1186/s13287-019-1195-z) contains supplementary material, which is available to authorized users.

## Background

Implants are being used to replace damaged or injured tissues and organs following trauma, cancer resection or congenital diseases. Such implants can be composed of autologous tissue or synthetic materials. In both cases, the survival of large tissue substitutes to replace damaged organs is reliant on the formation of an adequate blood supply [1, 2]. Angiogenesis is the process by which a new vascular network is formed. This structured and complex process involves the activation and movement of endothelial cells to form new blood vessels from pre-existing vasculature [1, 2].

Our group has been developing a synthetic polyurethane nanocomposite implant (PU) to replace the missing cartilage of large facial organs including the ear and nose due to congenital diseases or following cancer resection [3]. The material has proven to be biocompatible and support cell growth and tissue formation in vivo [4, 5]. One drawback of the polyurethane nanocomposite is that the material is hydrophobic, which prevents cell adhesion and consequential tissue integration and angiogenesis when implanted below the skin. Plasma surface modification (PM) is a technique, which can reverse the hydrophobicity of the implant surface. Radiofrequency plasma passes an electric current through a gas at low pressure [6, 7]. We have recently demonstrated that argon plasma surface modification can reverse the hydrophobicity of the polyurethane material and etch and clean the biomaterial surface [8]. The modified surface has shown to support fibroblast adhesion, growth, formation of extracellular matrix (ECM) and tissue integration and angiogenesis in vivo [8]. Argon plasma was more effective than nitrogen and oxygen plasma surface modification in promoting tissue integration and angiogenesis in vivo after 3 months in a rodent model [6]. However, little vessel formation was observed at 6 weeks in all the constructs. For future up scaling of the polyurethane implants as a large tissue construct to replace the cartilage of an ear and nose, we need to optimise further the angiogenesis potential of the polyurethane to ensure maximal survival of the implant.

Multiple techniques have been used to improve the angiogenic potential of implants following implantation [6]. It has been postulated that providing a source of angiogenic growth factors such as vascular endothelial growth factor (VEGF) or platelet-derived growth factor (PDGF) to scaffolds may improve the angiogenesis of implants in vivo. Platelet-rich plasma (PRP) is being investigated as a source of angiogenic growth factors for tissue engineering applications [9]. The definition of PRP is plasma with a platelet concentration of more than 1.0–10^6^ cell/μl or four to eight times greater than normal plasma [9]. Animal studies have shown the benefits of using PRP for improving the angiogenesis of implants. PRP has demonstrated to improve the limb perfusion in hindlimb and improve wound healing of cutaneous wounds and bone formation in defect models [10–12].

The application of mesenchymal stem cells (MSCs) to implants is another approach, which can improve the vascularisation of tissue-engineered constructs. Angiogenesis has been improved in the ischaemic setting by MSCs via the secretion of growth factors such as VEGF [13]. MSCs also have the ability to differentiate into vascular cells to produce vessels and form a vascular network [14]. One particular population of adult MSCs widely used to improve the angiogenesis of tissue-engineered constructs are adipose-derived stem cells (ADSCs) [15]. ASDCs are isolated from adipose tissue and have certain advantages when compared to bone marrow-derived stem cells. ADSCs can be harvested with a high yield from adipose tissue using non-invasive procedures compared to bone marrow stem cells [14]. ADSCs have shown to secrete angiogenic growth factors including vascular endothelial growth factor (VEGF) and basic fibroblast growth factor (bGFG) to contribute to the angiogenesis of scaffolds [14, 16]. Due to their angiogenic properties and ease of isolation, ADSCs have been widely investigated for improving the angiogenesis of tissue-engineered scaffolds [14].

PRP and ADSCs have also been used together to improve angiogenesis and improve tissue regeneration of scaffolds [17, 18]. The biological capacity of PRP is still in its infancy, but PRP has shown to improve ADSC differentiation [17, 18]. A few clinical studies have shown the beneficial effect of PRP and ADSC [17, 18]. Injection of PRP and ADSCs were used to treat osteoarthritis in 91 patients [17]. Furthermore, a combination of PRP and ADSC was effective in reducing pain and improving knee function in 18 patients [18]. However, the optimal number of ADSCs seeded on the scaffolds and PRP concentration to promote tissue integration and angiogenesis is varied among studies. Studies to date have used between 1 × 10^5^ and 2 × 10^5^ ADSCs when used in combination with PRP to improve the angiogenesis of scaffolds [19–21]. Further evidence and understanding is needed to evaluate the use of PRP and ADSCs on improving the therapeutic angiogenesis of tissue-engineered scaffolds.

When scaffolds are modified with biological functionalization such as PRP and ADSCs to improve the angiogenesis of the implant, it is important to determine the inflammatory response of the treatment with the surrounding tissue. The inflammatory response to an implant is a complex process involving multiple cell types [22]. The macrophage is the key inflammatory cell, which determines the inflammatory cell migration as well as controls the balance between tissue regeneration and tissue rejection [22].

We propose that the addition of platelet-rich plasma (PRP) and adipose-derived stem cells (ADSCs) to an optimised argon-modified biomaterial will improve the angiogenesis and tissue integration of the implant following implantation. The aim of this study was to isolate rat ADSCs and PRP to ascertain their ability to improve tissue integration and angiogenesis of nanocomposite polyurethanes in combination with argon plasma surface modification in vivo whilst monitoring for any signs of an inflammatory reaction. We demonstrate that argon plasma surface modification can promote the effect of ADSCs on tissue integration and angiogenesis of tissue-engineered scaffolds.

## Materials and methods

### Fabrication of polyurethane nanocomposite scaffolds

The polyurethane nanocomposite scaffolds (PU) were synthesised and fabricated as previously described by Griffin et al [3, 23]. In brief, polycarbonate polyol (2000 Mwt) and transcyclohexanechloroydrinisobutyl-silses-106 quioxane (Hybrid plastics Inc.) were mixed under a nitrogen environment in a 500-ml flask containing a stirring rod. The POSS cages were then dissolved into the polyol solution at 70 °C. Following this, 4,4-methylenebis (phenyl 109 isocyanate) MDI was added to the solution to form a pre-polymer. Then, dimethylacetamide (DMAC) was added slowly to the pre-polymer to create a polymer solution. Chain extension was then performed by the addition of ethylenediamine and diethylamine in DMAC slowly. The scaffolds were fabricated by a porogen leaching/solvent casting technique. The POSS-modified polycarbonate urea-urethane polymer solution was mixed with sodium chloride porogen (NaCl with pore size 150–250 μm) in a ratio of 1:1. The slurry solution was mixed and degassed in the Thinky AER 250 mixer (Intertronics, Kidlington, UK). The slurry was then coated onto titanium moulds and left in an air circulating oven at 65 °C for 4 h. The PU sheets were then submerged into deionised water, for 24 h. This process was repeated until the required thickness was achieved. The scaffolds were then further washed to remove NaCl and DMAC completely. The scaffolds were then cut into 15-mm diameter discs for in vitro and in vivo experiments. Argon modification of PU scaffolds was performed by exposing the scaffolds to 5 min using a radiofrequency plasma generator operating at 40 kHz with gas flow of 0.4mbar at 100 W. Plasma-modified scaffolds were sterilised prior to argon plasma modification using a standard autoclaving protocol. Scaffolds without argon modification were also autoclaved prior to use and referred to as PU.

### Rat adipose-derived stem cell (rADSC) isolation and characterisation

For in vivo experiments, allogenic rat ADSCs were isolated from the epididymal fat pads of 12-week-old male Sprague-Dawley rats. ADSCs were isolated according to the method described by Naderi et al [24]. In brief, following removal of fibrous tissue and visible blood vessels, samples were cut into small pieces (< 3 mm^3^) and digested in Dulbecco’s modified Eagle’s medium/nutrient mixture F-12 ham (DMEM/F12) containing 300 U/ml crude collagenase I (Invitrogen, Life Technologies Ltd, Paisley, UK) for 30 min in an incubator (37 °C, 5% CO_2_). Subsequently, 10% foetal bovine serum (FBS) was added to the dispersed material and filtered through 70-μm cell strainers (BD Biosciences, Oxford, UK). After centrifugation (290×*g* for 5 min), the supernatant was removed and the ADSC-containing pellet re-suspended. The number of viable cells was determined by cell counting on a haemocytometer and trypan blue exclusion. Cells were cultured for up to two passages DMEM/F12 supplemented with 10% FBS and 1% penicillin solution. At each subsequent passage, cells were seeded to sub-confluence in 75-cm^2^ culture flasks for 7 to 8 days at a cell density of 3 × 10^4^/cm^2^. When the cells reached approximately 80% confluence, subculture was performed through trypsinisation. The cell suspension was centrifuged (290×*g* for 5 min), the pellet was re-suspended and cells were counted as before and then plated. Passage 2 rADSCs were seeded on the polymer discs for in vitro analysis.

ADSCs from passage 0 were immunophenotypically characterised using flow cytometry (*n* = 3) as described by Naderi et al [24]. In brief, ADSCs were stained with antibodies for different CD (cluster of differentiation) antigens. Additional file 1: Table S1 lists the different antibodies, their fluorochrome, emission/excitation wavelength, clone, isotype and dilution. For analysis, 1 × 10^6^ cells at passage 0 per flow cytometry tube were suspended in 0.2 ml PBS and incubated with the antibodies for 30 min on ice and protected from light. The samples were acquired using flow cytometry (MACSQuant® Analyser 10; Miltenyi Biotec, Cologne, Germany) with machine settings as detailed in Additional file 2: Table S2. Kaluza software (version 1.2; Beckman Coulter, USA) was used to analyse the characterisation data.

### Preparation of PRP

Platelet-rich plasma (PRP) preparation was performed as previously described with modification [25]. Firstly, 20 ml of whole blood from allogenic 12-week-old Sprague-Dawley rats was drawn percutaneously from the heart at the time of termination into tubes containing 3.8% sodium citrate. The tubes containing blood were centrifuged for 40 min at 200×*g*. The buffy coat, which contains PRP, in between the supernatant plasma and white blood cell layer, was collected into a neutral tube with a long pipette. PRP gelation was activated with a 10% calcium chloride solution and thrombin immediately before administration in vivo. An automated platelet counter showed that the platelet concentration in the PRP was 20.2 × 10^4^/ml, tenfold higher than the rat blood.

### Experimental design

Polyurethane scaffolds without argon modification were included as the control group for this study and referred to as PU. The PU scaffolds modified with argon plasma surface modification were referred to as PM scaffolds. For the ADSC group, 1 × 10^6^ P2 ADSCs were seeded onto each scaffold. The scaffolds were then incubated for 24 h prior to implantation and referred to as ADSC scaffolds. The incubation period of 24 h allowed for adequate adhesion of the ADSCs to the scaffolds. For the PRP group, 1 ml of activated PRP at a concentration of 20.2 × 10^4^/ml (tenfold higher than that of normal plasma) was coated on the scaffold for 30 min prior to implantation and referred to as PRP scaffolds. At 6 and 12 weeks post implantation, the rats were sacrificed by CO_2_ overdose and the scaffolds were explanted within the surrounding tissues for histological and immunohistochemistry analysis. A brief overview of the scaffold and their modifications is demonstrated in Table 1.

**Table 1:** The scaffold modifications used in this study

Scaffold Group Number	Scaffold Type	Scaffolds Modification Technique	Abbreviation
1	Control Scaffolds	Polyurethane nanocomposite scaffolds without modification	PU
2	Scaffold modified with ADSCs	Polyurethane nanocomposite scaffolds modified with 1 x 10^6^ cells per scaffold. ADSCs seeded on the scaffold for 24 hours prior to implantation.	ADSC
3	Scaffold modified with PRP	Scaffold modified with PRP at a concentration of 20.2 x 10^4^/ml with a 30 minute incubation time prior to implantation.	PRP
4	Scaffolds modified with plasma surface modification	Scaffold modified with argon plasma surface modification for 5 minutes immediately prior to implantation.	PM
5	Scaffolds modified with plasma surface modification + ADSC	Scaffolds modified as per group 2 and 4.	PM + ADSC
6	Scaffolds modified with plasma surface modification + PRP.	Scaffolds modified as per group 3 and 4.	PM + PRP
7	Scaffolds modified with ADSC + PRP.	Scaffold modified with 2 + 3.	ADSC + PRP
8	Scaffolds modified with plasma surface modification + PRP + ADSC	Scaffolds modified as per group 2 and 3 and 4.	PM + PRP + ADSC

### Animals

All animals were treated with procedures approved by the local governmental animal care committee (University College London University, PL 70/7504), and experiments were conducted in accordance with the UK legislation on the protection of animals and the guidelines for the Care and Use of Laboratory Animals. For the implantation surgeries, male Sprague-Dawley rats were anesthetised with 2% isoflurane in 2 L/min of O_2_ and the incision site was marked with povidone-iodine. A 1-cm incision was made in the dorsal dermis of the rats, and the scaffolds were carefully positioned in the subcutaneous space. The wounds were closed with 5/0 Monocryl dermal and subcuticular sutures. Each rat received two implants, and all scaffold types were assessed equally (*n* = 6) at each time point. No adverse events were noted with any of the animals. During the experiments, the animals were housed in groups and had free access to water and pellet food.

### In vitro assessment

The adhesion, proliferation and angiogenic response of the P2 rADSCs to scaffolds modified by the addition of PRP (PRP), argon-modified scaffolds (PM) and the combination of PRP on argon-modified scaffolds (PRP+PM), was assessed prior to in vivo implantation. The culture medium for the PRP and PRP+PM scaffolds were changed to 10% PRP instead of 10% FBS for these experiments.

#### Rat adipose-derived stem cell morphology

The morphology of the rADSCs was assessed using F-actin staining after PRP and PM modification of the PU scaffolds as previously described [3, 23] after 6 h of attachment. In brief, 15,000 cells were seeded onto the scaffolds for assessment of rADSC stem cell morphology. The media was then removed from the 24 wells at 6 h. The cells were washed with PBS several times and then fixed with 4% (*w*/*v*) paraformaldehyde in PBS pre warmed at 37 °C for 10–15 min. Following washing with 0.1% Tween 20 thrice and incubation with 0.1% TritonX-100 for 5 min to improve permeability, the cells were stained with rhodamine-conjugated phalloidin (ThermoFisher Scientific, UK) in the ratio 1:40 (stock 1: 1000 in methanol) for 40 min. Following washing, the cells were stained with DAPI (4′,6-diamidino-2-phenylindole, 1:500) to stain the nuclei. The cells were visualised with a confocal laser-scanning microscope (LSM 710, Zeiss). The cells were analysed using ImageJ Software 1.48V (National Institute of Health USA) to determine cell circularity and cell size (the surface occupied by spread of the actin cytoskeleton). A total of 30 cells were analysed on six scaffolds, taking an average for comparison (*n* = 6).

#### Rat adipose-derived stem cell growth

The adhesion and growth of the rADSCs were assessed on the PU scaffolds using DNA quantification as previously described [3, 23]. In brief, DNA content was assessed after 1, 2, 4, 7 and 14 days of in vitro culture using the Fluorescence Hoechst DNA quantification kit (Sigma, UK) performed according to the manufacturer’s instructions (*n* = 6).

#### Angiogenesis of rat adipose-derived stem cells

The angiogenic response of the rADSCs was assessed using three methods in vitro.

##### Quantification of the secretion of the angiogenic growth factors using ELISA

Firstly, the angiogenic response of the rADSCs was assessed by quantification of angiogenic growth factors, vascular endothelial growth factor (VEGF), basic fibroblast growth factor (bFGF) and hepatocyte growth factor (HGF), using sandwich enzyme-linked immunosorbent (ELISA; Quantikine, R&D System, Abingdon, UK) on days 7 and 14 of culture (*n* = 6).

##### Immunocytochemistry staining of vascular endothelial growth factor (VEGF)

After 14 days in culture, the scaffolds were washed in PBS and then fixed in 4% PFA overnight at 4 °C. Following washing in PBS, the scaffolds were permeabilised (0.5% Triton X-100 in PBS) and blocked in 0.5% BSA in PBS for 1 h at room temperature. Sections were incubated with primary antibodies diluted in blocking solution overnight at 4 °C (abcam ab1316, UK, 1:200 dilution). After washing in PBS, the scaffolds were incubated with secondary antibodies for 2 h at room temperature (Alexa Fluor 488, 1:500). Following staining of the cell nuclei with Hoechst 33258 (2.5 μg/ml final concentration), the scaffolds were digitally scanned using a confocal laser-scanning microscope (LSM 710, Zeiss) (*n* = 6).

##### Angiogenesis by RT-qPCR of the rADSCs

RNA was extracted from the scaffolds after 14 days using Tri-Reagent (Life Technologies) according to the manufacturer’s instructions to examine VEGF and BFGF expression and performed as previously described [23]. The RNA was reverse-transcribed with Moloney murine leukaemia virus reverse transcriptase (Promega, Madison, WI). Primer sequences and annealing temperatures for VEGF primer were F: CCCACTGAGGAGTCCAACAT and R: TTTCTTGCGCTTTCGTTTTT; for BFGF were F: AGAACGGCGGCTTCTTCCT and R: CCCTTGATGGACACAACTCC; and for housekeeping gene GAPDH were F: ATGTGCCGGACCTTGGAAG and R: CCTCGGGTTAGCTGAGAGATCA. Real-time quantitative polymerase chain reaction (qPCR) was performed with an ABI Prism 7500 sequence detection system (Applied Biosystems) using the QuantiTect SYBR Green PCR kit (Qiagen, Hilden, Germany) according to the manufacturer’s instructions. Gene expression data were normalised using GAPDH housekeeping gene as a reference using the 2-ΔΔCt method (*n* = 6).

### In vivo analysis

#### Immunohistochemistry

After 6 and 12 weeks, the animals were sacrificed by CO_2_ asphyxiation and the scaffolds were explanted, fixed in 4% paraformaldehyde and analysed for tissue integration, angiogenesis and immune response as previously described [3]. The scaffolds were embedded in paraffin, and 3-μm sections were cut and stained with H&E and Masson’s trichrome according to standard procedures. The Masson’s trichrome in this study illustrated collagen by green staining. In addition, scaffolds were stained against CD31 to detect endothelial cells (Abcam 28364, UK, 1:50 dilution), CD45 (Abcam 10558, UK, 1:20 dilution) and CD68 (Abcam 125212, UK, 1:150 dilution) to detect leucocyte and macrophages respectively. The sections were then imaged using a digital slide scanner, NanoZoomer-XT C12000, Hamamatsu Photonics. To quantify the extent of cellular integration into the scaffold, 5 fields of view (× 40 magnification) were chosen at random and the percentage of tissue infiltrated in the view was divided by the tissue stained by H&E staining to formulate the percentage of cellular integration. To quantify vessel formation, the methodology used was as per a previous study [3, 26, 27]. Briefly, as the scaffold did have some background staining, the capillary number was calculated by identifying a positive endothelial cell cluster with a morphologically identifiable vessel with a lumen in 5 fields of view at × 40 magnification on each scaffold, providing 20 fields of view in total. To quantify the number of positive CD45 and CD68 cells, 3 fields of view (× 40 magnification) were chosen at random to count the number of positive cell per square micrometer using NDP Hamamatsu software analysis. As the scaffolds did have some background staining, the CD45 and CD68 cells were identified as cells with positive staining within the scaffold tissue and not the scaffold itself.

#### Assessment of renal and hepatic toxicity

Blood samples were taken from the rats at 0, 6 and 12 weeks, which were implanted with PU scaffolds to assess their renal and liver function throughout the study. Blood samples were obtained from the tails under aseptic techniques and examined for full blood count (FBC), white cell count (WCC), urea, creatinine and liver function (AST, ALT and ALP). Blood tests were taken from all three rats at each time point, and an average was calculated for evaluation (*n* = 3).

## Results

### In vitro assessment

The adhesion, proliferation and angiogenic response of the rADSCs to the addition of PRP and PM to PU scaffolds was first assessed in vitro. The rADSCs were characterised and stained with CD44+/CD34+/CD90+/CD45−/CD31 using flow cytometry analysis (Additional file 3: Figure S1).

#### Rat adipose-derived stem cell morphology, adhesion and growth

**Fig. 1:**
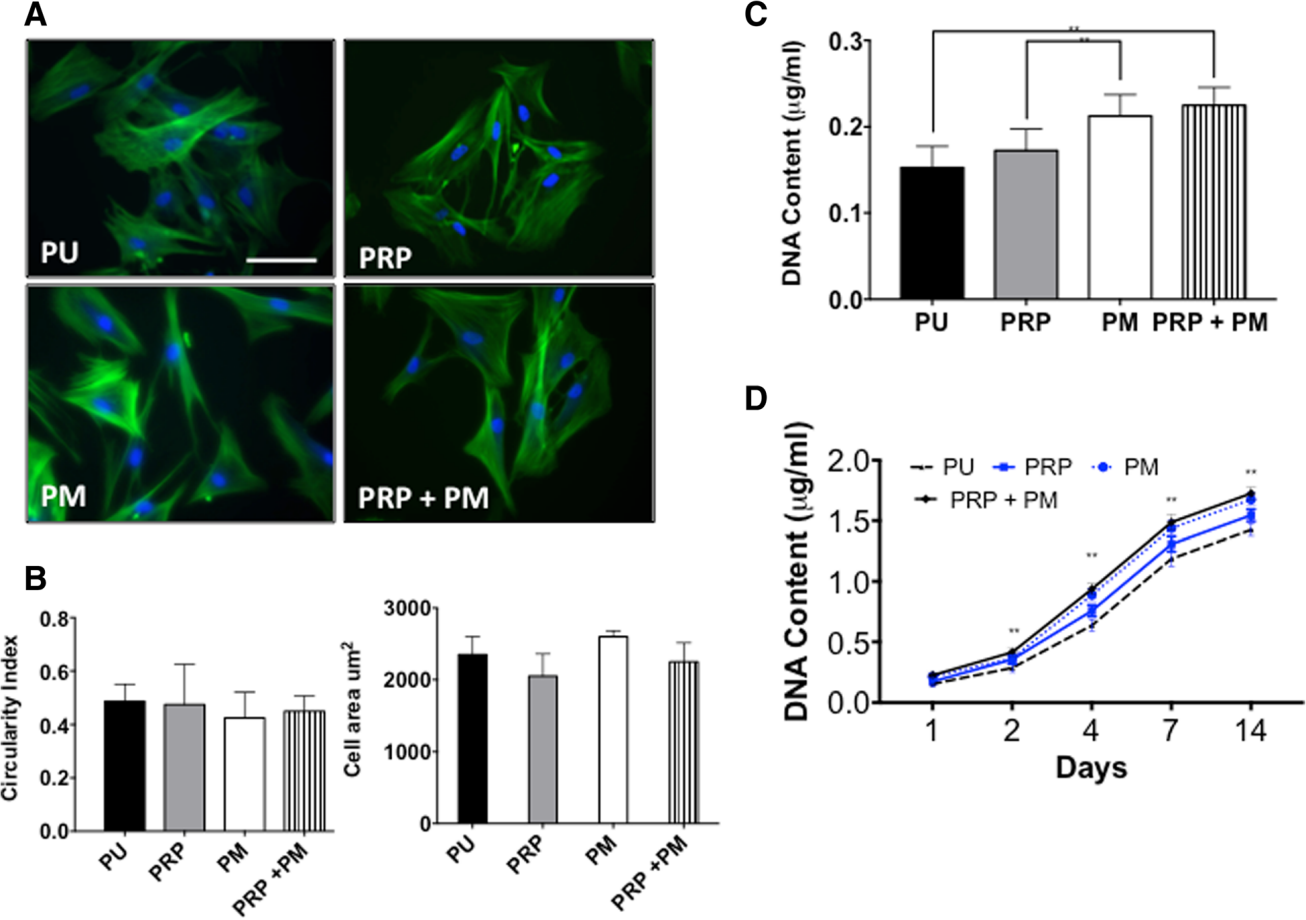
Evaluation of the rat adipose-derived stem cell (rADSC) adhesion and cell morphology on the modified scaffolds. **a** Cell morphology of the rADSCs after 6 h on the modified scaffolds by staining F-actin. Green indicates phalloidin, and blue indicates nucleus staining using DAPI. Scale bar refers to 100 μm. **b** Quantification of the F-actin morphology including cell circularity and cell area quantification showed no difference in cell circularity between the scaffolds. **c** Adhesion of the rADSCs after 24 h using DNA content assay. Note the PM+PRP and PM scaffolds showed significantly greater adhesion after 24 h compared to the PU and PRP scaffolds (**p < 0.01). **d** DNA content of the rADSCs after 14 days on the modified scaffolds. Note the PM+PRP and PM scaffolds showed significantly greater adhesion at days 2, 4, 7 and 14 compared to the PU and PRP scaffolds (**p < 0.01). PU unmodified scaffolds, PRP platelet-rich plasma-modified scaffolds, PM argon-modified scaffold, PRP+PM platelet-rich plasma and argon modification (*n* = 6)

F-actin stained the morphology of the rADSCs after adhering to the PRP and argon-modified scaffolds after 6 h (Fig. 1a). There were no changes in the cell shape of the rADSCS seeded among the different scaffolds after 6 h as demonstrated by ImageJ analysis (Fig. 1b). The adhesion of the rADSCs to the PM and PRP+PM was significantly greater than PRP and unmodified PU control scaffolds (*p* < 0.05). PRP did not significantly improve the adhesion of the rADSCs compared to unmodified control scaffolds (Fig. 1c). All scaffolds allowed for rADSC growth over the 14-day period (Fig. 1d). The rADSC DNA content on the PM+PRP scaffolds showed the highest growth at each time point, which was significantly greater than PRP and PU (*p* < 0.05) but not significantly different to PM scaffolds. The addition of PRP to the scaffolds demonstrated higher DNA content from days 2–14 compared to the control scaffolds (*p* < 0.05).

#### Secretion of angiogenic factors

**Fig. 2:**
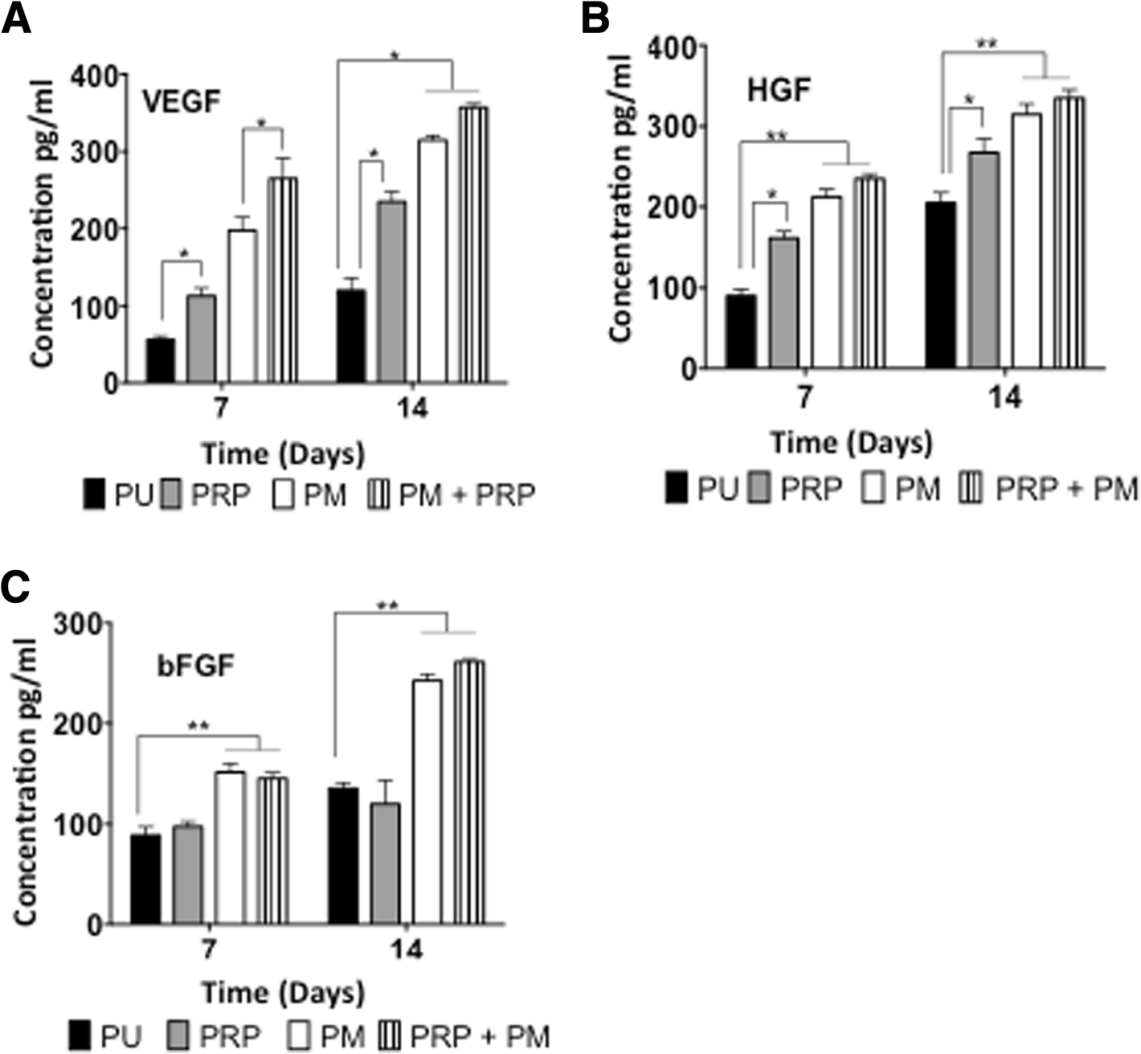
ELISA analysis of the secretion of the angiogenic factor secreted by the rat adipose-derived stem cells (rADSCs) on the modified scaffolds. **a** Vascular endothelial growth factor (VEGF), **b** hepatocyte growth factor (HGF) and **c** basic fibroblast growth factor (bFGF) secretion by the rADSCs after 7 and 14 days (*n* = 6). Note that rADSCs on the PRP+PM and PM scaffolds secreted significantly more angiogenic factors including VEGF, HGF and bFGF than those on the PU and PRP scaffolds (*p* < 0.05). PU unmodified scaffolds, PRP platelet-rich plasma-modified scaffolds, PM argon-modified scaffold, PRP+PM platelet-rich plasma and argon modification. *p* values, *p< 0.05 and **p< 0.01

The release of angiogenic growth factors VEGF, bFGF and HGF was quantified to determine the angiogenic response of the rADSCs at days 7 and 14. The rADSCs grown on PM and PM+PRP scaffolds secreted significantly greater levels of VEGF, HGF and bFGF than those growing on unmodified and PRP scaffolds (*p* < 0.05) (Fig. 2a–c). VEGF and bFGF secretion by the rADSC was significantly higher than unmodified scaffolds (*p* < 0.05), but the secretion of HGF was similar. The VEGF secretion by the rADSCs on the PRP+PM was significantly higher than that compared to the PM (*p* < 0.05), but the secretion of HGF and bFGF showed no difference.

#### Gene expression of angiogenesis

The expression of angiogenic marker VEGF was determined by RT-qPCR to determine the angiogenic response (Additional file 4: Figure S2). The mRNA expression of VEGF was also significantly greater on the PM and PM+PRP scaffolds than on the unmodified scaffolds (*p* < 0.05). PRP modification also increased the expression of angiogenic-related genes by the rADSCs at 14 days compared to the unmodified PU (*p* < 0.05). There was no significant difference in the angiogenic expression by the rADSCs on the PRP+PM scaffolds compared to the PM scaffolds. Immunocytochemistry of VEGF also confirmed the RT-qPCR findings with greater staining on the PM and PRP+PM scaffolds (Additional file 4: Figure S2).

### In vivo analysis

#### Tissue integration

**Fig. 3:**
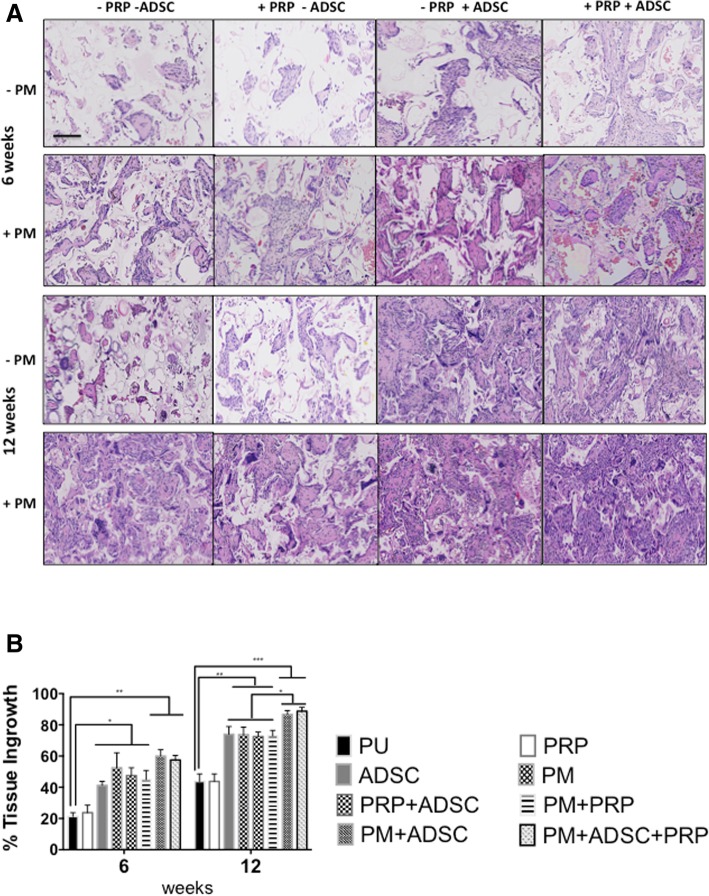
Tissue integration of the scaffolds treated with platelet-rich plasma (PRP), plasma surface modification (PSM) and rat adipose-derived stem cells (rADSCs) at 6 and 12 weeks using H&E analysis. **a** H&E analysis. Scale bars refer to 400 μm. **b** Quantification of tissue integration analysis of the modified PU scaffolds after 3 months of subcutaneous implantation in a rodent model (*n* = 6). Note that the PM+ADSC and PM+ADSC+PRP scaffolds showed significantly greater tissue integration than all other scaffolds (*p* < 0.05). PU unmodified scaffolds, PRP platelet-rich plasma-modified scaffolds, PM argon-modified scaffold, ADSC rat adipose-derived stem cell-modified scaffolds. *p values < 0.05, **p < 0.01 and *** *p* < 0.001

**Fig. 4:**
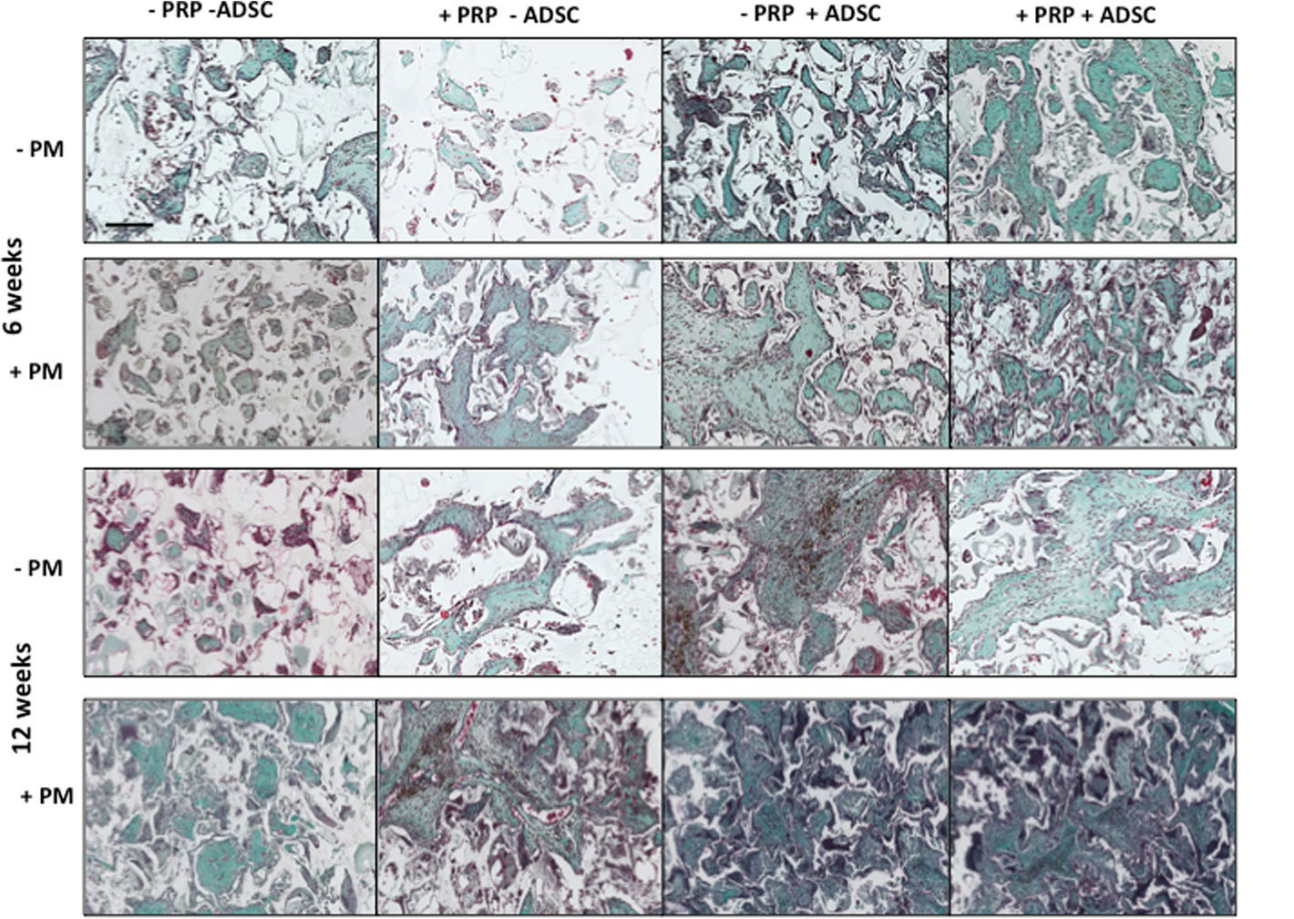
Masson’s trichrome staining at 6 and 12 weeks of subcutaneous implantation of the scaffolds treated with platelet-rich plasma (PRP), plasma surface modification (PSM) and rat adipose-derived stem cells (rADSCs). Note that the PM+ADSC and PM+ADSC+PRP scaffolds showed greater collagen staining than all other scaffolds over the 12 weeks (*n* = 6). Scale bars refer to 400 μm. PU unmodified scaffolds, PRP platelet-rich plasma-modified scaffolds, PM argon-modified scaffold, ADSC rat adipose-derived stem cell-modified scaffolds

Tissue integration was assessed by H&E and collagen formation using Masson’s trichrome. Over 12 weeks, PU and PRP of the eight groups showed similar levels of tissue integration, which was the least integrated, compared to the other scaffolds examined (*p* < 0.05) (Fig. 3a). PM, ADSC, PM+PRP and ADSC+PRP demonstrated similar levels of tissue integration, which was higher than PU and PRP (Fig. 3b). The two scaffolds, which showed the greatest integration compared to other groups, were PM+PRP+ADSC and PM+ADSC, but there was no significant difference between the two scaffolds (Fig. 4). Masson’s trichrome staining confirmed the elevated tissue integration seen with H&E staining, with greater collagen in the PM+PRP+ADSC and PM+ADSC scaffolds (Fig. 4).

#### Angiogenesis

**Fig. 5:**
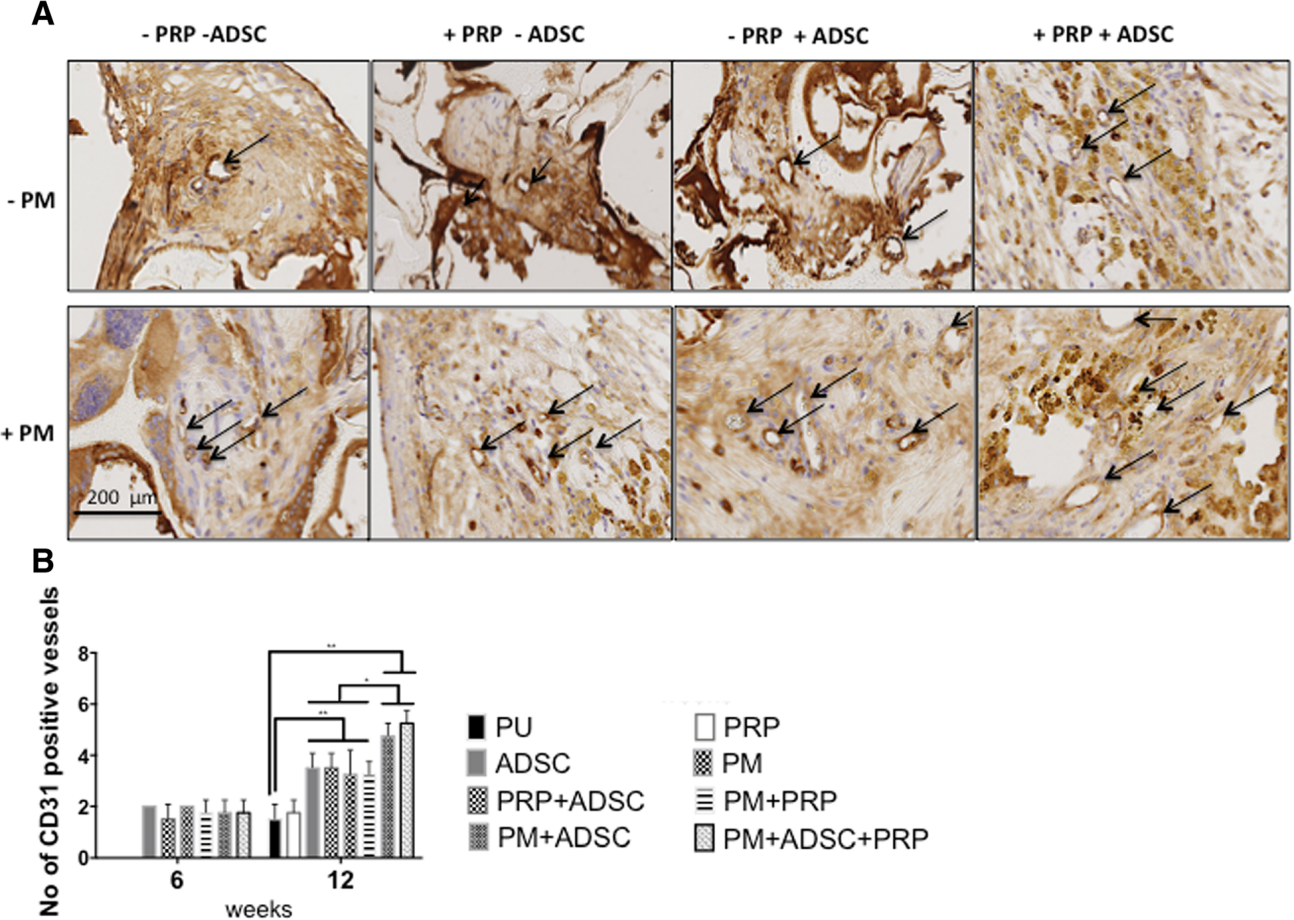
Angiogenesis analysis of the scaffolds over 12 weeks. **a** Angiogenesis assessment of the scaffolds treated with platelet-rich plasma (PRP), plasma (PM) and rat adipose-derived stem cells (rADSCs) at 12 weeks as shown by CD31 staining (*n* = 6). **b** Quantification of the number of positive CD31 cells at 6 and 12 weeks. Note that PM+ADSC and PM+ADSC+PRP scaffolds have significantly greater vessel numbers than all other scaffolds over the 12 weeks (*p* < 0.05). PU unmodified scaffolds, PRP platelet-rich plasma-modified scaffolds, PM argon-modified scaffold, PRP+PM platelet-rich plasma and argon modification. *p* values *< 0.05, **p < 0.01 and ***p < 0.001. Arrows illustrate positive CD31 stained vessels

The angiogenesis response was evaluated by staining the tissue with CD31 to examine vessels (Fig. 5a, b). After 12 weeks, PU control and PRP showed similar levels of vessel formation, which was the lowest compared to the other scaffolds (*p* < 0.05). PM, ADSC, PM+PRP and ADSC+PRP demonstrated similar levels of vessel formation, which was significantly higher than PU control and PRP over 12 weeks. The two scaffolds, which showed the greatest vessel formation compared to other scaffolds, were PM+PRP+ADSC and PM+ADSC, but there were no significant differences between the two scaffolds after 6 or 12 weeks.

#### Immune response

**Fig. 6:**
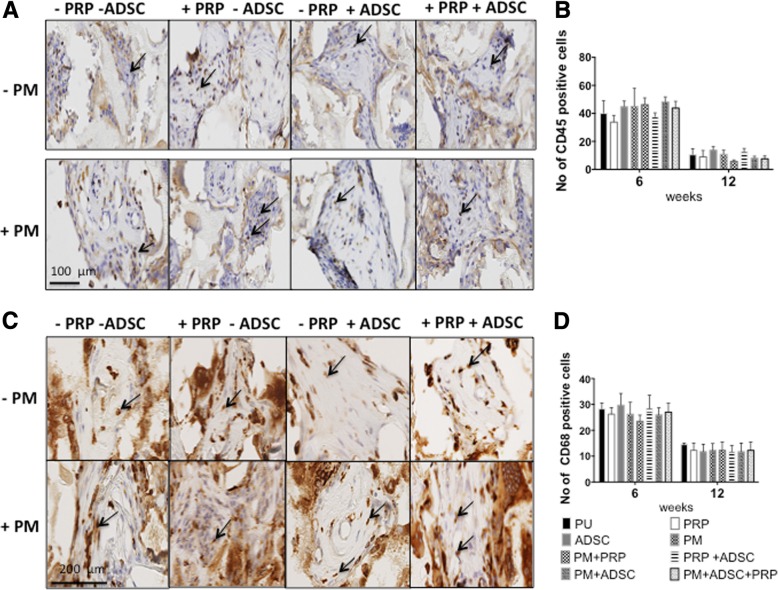
Immune response of the scaffolds over the 12 weeks. **a** CD68 macrophage assessment of the scaffolds treated with platelet-rich plasma (PRP), plasma (PM) and adipose-derived stem cells (ADSCs) at 12 weeks (*n* = 6). **b** Quantification of macrophage infiltration at 6 and 12 weeks. Note that there was no significant difference between the CD68 staining among the scaffolds. **c** CD45 leucocyte assessment of the scaffolds treated with platelet-rich plasma (PRP), plasma (PM) and adipose-derived stem cells (ADSCs) at 12 weeks (*n* = 6). **d** Quantification of CD45 leucocyte infiltration at 6 and 12 weeks. Note that there was no significant difference between the CD45 staining among the scaffolds. PU unmodified scaffolds, PRP platelet-rich plasma-modified scaffolds, PM argon-modified scaffold, PRP+PM platelet-rich plasma and argon modification. Arrows illustrate positive CD45 and CD68 stained cells

Staining of the scaffolds with CD45 and CD68 assessed the immune response of the scaffolds (Fig. 6). The scaffolds showed a similar decrease in CD45 from 6 to 12 weeks with no difference between the scaffolds. The infiltration of cells expressing CD68 was also similar between all the scaffolds from 6 to 12 weeks.

### Assessment of haematological, renal and hepatic toxicity

It was important to evaluate the blood serum levels to ensure the rats were not showing signs of infection or failure due to the addition of the PRP and ADSC to the scaffolds. The blood test at 0, 6 and 12 weeks showed no change in haematological levels (RBC, WBC) or renal or liver markers for any of the scaffolds (urea/creatinine/AST/ALT/ALP) (Additional file 5: Figure S3).

## Discussion

This study has examined the effect of argon plasma surface modification, PRP and ADSCs on the tissue integration, angiogenesis and immune response of synthetic scaffolds. This study provides evidence that ADSCs can promote the vascularisation of synthetic scaffolds but PRP has little effect. Argon surface modification promoted the angiogenic and tissue formation effect of ADSCs on the nanocomposite scaffolds.

The data showed there were three levels of tissue integration and angiogenesis of the synthetic materials over the 12-week period. The least integrated scaffolds with the lowest number of blood vessels were control scaffolds and scaffolds modified with PRP alone over the 12 weeks. The second level was the PM, ADSC, PM+PRP and ADSC+PRP scaffolds which all showed a similar level of integration and angiogenesis. The highest level of integration and blood vessel formation was observed with the PM+PRP+ADSC and PM+ADSC scaffolds.

This data demonstrates that ADSCs and plasma surface modification were enhancers of tissue integration and angiogenesis whereas PRP had no effect. Scaffolds modified with PRP demonstrated no effect on tissue integration and angiogenesis compared to control but enhanced the tissue integration and angiogenesis when PM scaffolds or ADSCs were also present. Modification of the scaffolds with ADSCs or PM demonstrated improvements in angiogenesis and tissue integration of the synthetic scaffolds. When both ADSC and PM scaffolds were present, the scaffold modifications acted synergistically, indicating that the argon plasma surface modification promoted the ADSCs on angiogenesis and tissue integration. Additional file 6: Figure S4 schematically explains these key findings.

It was important to understand the effect of argon plasma and PRP on rADSC growth, adhesion and angiogenic properties in vitro as well as in vivo environment. The in vitro data supports the in vivo findings that PM was a more effective surface modification than PRP in promoting tissue formation and angiogenesis. Plasma surface modification improved the rADSC adhesion and growth in culture to a greater degree than scaffolds treated with PRP alone (Fig. 1). Furthermore, plasma surface modification improved the secretion of angiogenic growth factors and mRNA expression of VEGF of the rADSCs to a greater degree than on scaffolds treated with PRP alone (Additional file 4: Figure S2). Both plasma surface modifications and PRP caused no change in the cell shape and cell area of the rADSCs as shown by F-actin staining adhering to the scaffolds, providing evidence that the modifications provided suitable sites for adhesion (Fig. 1).

PRP did improve the growth of the rADSCs on the PU scaffolds, as in agreement with previous studies [28–30]. Diaz et al demonstrated PRP can improve the adhesion and proliferation of ADSCs on PCL scaffolds [30]. PRP has also shown to increase the secretion of angiogenic factors by ADSCs in vitro culture [28–30], which explains the increased secretion on the PRP scaffolds, compared to the unmodified control. Several reports have shown that PRP alone can increase the angiogenesis of scaffolds in vivo [31–38]. The use of PRP on poly(lactic-co-glycolic acid) (PRP-PLGA)/calcium phosphate cement (CPC)PLGA/CPC composite scaffolds improved the bone formation, angiogenesis and material degradation after 12 weeks of implantation in a rabbit defect model [34]. The addition of PRP to strattice in an in vivo ventral hernia repair model showed enhanced neovascularisation in a rat model over 6 weeks [35]. Our study is in contrast with the current literature, as PRP was found to have no significant effect of tissue integration and angiogenesis when used alone on the polyurethane scaffolds. However, despite the pre-clinical experiments providing some evidence that PRP may have a therapeutic effect, the clinical evidence is minimal [36].

The observation that PRP has little effect on the angiogenesis in this study may be due to several factors. We fixed the concentration of PRP based on previous studies and preliminary studies, which showed rADSC adhesion and viability and VEGF and bFGF expression over 14 days in vitro was optimal with concentration with 30-min incubation (Additional file 7: Figure S5). Although the concentration of PRP in vitro caused significant changes to the rADSC angiogenic potential, the in vivo setting is more complex to control. There are multiple cues and processes that the PRP might need to influence to improve tissue integration and angiogenesis.

Few studies have shown that PRP and ADSCs work synergistically together to improve tissue integration and angiogenesis [39–41]. For example, ADSCs with PRP have shown better wound healing after radiation treatment than hADSCs alone in a porcine model [37]. Furthermore, Seyhan et al. reported that fat grafts were more viable in a mouse model with ADSCs and PRP than when used alone [36]. Chiou et al. also found that ADSCs and PRP improved tendon healing when used together due to the release of angiogenic growth factors [39]. However, in this study, there was no enhancing effect of ADSC and PRP as a similar level of tissue integration and angiogenesis was observed with PU+ADSC and PU+ADSC+PRP using histological analysis. However, this study did provide evidence that plasma-modified scaffolds worked synergistically with the rADSC on improving the angiogenesis and tissue integration of the nanocomposite polyurethane scaffolds.

We have shown that argon creates a hydrophilic surface by depositing hydroxyl groups on the implant surface [3]. The argon modification may have produced optimal topographical and surface chemical changes to allow for optimal rADSC adhesion, which consequently supported tissue integration and angiogenesis in vivo. Few studies have shown that plasma surface modification may improve the angiogenesis of synthetic materials [40]. Ring et al. demonstrated that argon/hydrogen plasma improves the neovascularization of Matriderm [40]. Our group has also shown that plasma surface modification using polymerisation with carboxyl and amine groups can improve the angiogenesis of polyurethane implants over 3 months in a rodent study [20]. However, this study provides a single-step plasma surface modification technique, which allows for an easier clinical translation. Argon plasma surface modification is easily transferrable to other implant biomaterials as it only modifies the surface without altering the structural and mechanical properties of the implant [5, 6]. This study provides an easy tool to improve the therapeutic effect of ADSCs for tissue engineering applications.

After modifying an implant with cells or growth factors, there is a concern that the implant may then cause an inflammatory reaction. ADSCs have anti-inflammatory properties and used as treatment options in clinical conditions to decrease immune reactions [41–43]. PRP may also influence inflammatory reactions due to containing pro- and anti-inflammatory mediators [33]. The foreign body response is a process of events from inflammation, proliferation and tissue remodelling [2, 44]. In this study, there were similar levels of CD45-and CD68-positive cells among all scaffolds after 12 weeks, demonstrating a similar immune response. The CD68 macrophage is a complex dynamic cell type involved in both the immune and tissue remodelling responses of implants [45]. Recently, PRP and ADSCs have shown to improve the macrophage’s tissue regenerative capacity [45]. However, in this study, there were no significant changes in the implant response, suggesting the macrophage played a small role in modifying the tissue regeneration.

The data in this study suggests that the use of ADSCs to improve the angiogenesis and tissue integration should be considered. With current evidence showing varied number of cells improving angiogenesis, the optimal number is still unclear [19–21]. We provide evidence that 1 × 10^6^ is useful to improve angiogenesis of scaffold that are 15 mm wide and 500 μm thick. This information can be used for future up scaling of tissue-engineered constructs. However, future studies are needed to optimise the use of ADSCs with scaffolds for clinical application. For example, detailed analysis into the ADSC regenerative capacity when accounting for ages, genders and disease state must be performed [46–48].

## Conclusion

Argon surface modification promoted the ADSC effect on tissue integration and angiogenesis of subcutaneously synthetic implants. Despite previous studies demonstrating that PRP is useful to improve angiogenesis, this study found no evidence for this. This study supports the use of ADSCs in combination with argon surface modification to improve the vascularisation of large bioengineered scaffolds to improve the outcome of synthetic materials.

## Additional files

Additional file 1: **Table S1.** Details of flow cytometry antibodies including their fluorescent dye, excitation wavelength and dilution. FITC: fluorescein isothiocyanate, APC: allophycocyanin, PE: phycoerythrin. (DOCX 16 kb)

Additional file 2: **Table S2.** Flow cytometry voltage configurations. FSC: forward scatter, SSC: side scatter, FITC: fluorescein isothiocyanate, PE: phycoerythrin, APC: allophycocyanin. (DOCX 14 kb)

Additional file 3: **Figure S1.** Flow cytometry data of the rat adipose-derived stem cells seeded on the scaffolds. The rat adipose-derived stem cell (rADSCs) stained CD44+/CD34+/CD90+/CD45−/CD31. (TIFF 1521 kb)

Additional file 4: **Figure S2.** Analysis of angiogenesis using immunocytochemistry and RT-qPCR. [A] Immunocytochemistry of vascular endothelial growth factor (VEGF) from the rat adipose-derived stem cells (rADSCs) after in vitro culture on the modified scaffolds. Note that there was an increased expression of VEGF on PM and PM+PRP scaffolds. Green; VEGF, blue; DAPI. Scale bars 400 μm. [B] RT-qPCR analysis showed VEGF and basic fibroblast growth factor (BFGF) expression of the rADSCs after 14 days of culture. Note the significantly increased levels of expression of VEGF and BFGF on the PM and PRP+PM scaffolds compared to PU and PRP scaffolds (*p* < 0.05). Fold change is relative to housekeeping gene GAPDH of rADSCs grown on TCP. PU; unmodified scaffolds: PRP; platelet-rich plasma-modified scaffolds, PM; argon-modified scaffold. PRP+PM; platelet-rich plasma and argon modification. Sec; secondary antibody only. *p* values *< 0.05 and **< 0.01. (TIFF 1521 kb)

Additional file 5: **Figure S3.** Haematological and biochemistry blood test analysis of the animals over the 12 weeks following implantation of the different scaffolds. [A] Assessment of haematological function. [B] Assessment of liver function. [C] Assessment of renal function. Note no change in haematological, liver function or renal function following implantation of the scaffolds. PU unmodified scaffolds, PRP platelet-rich plasma-modified scaffolds, PM argon-modified scaffold, PRP+PM platelet-rich plasma and argon modification. (ZIP 84 kb)

Additional file 6: **Figure S4.** A schematic summary of the effect of PRP and ADSCs on tissue integration and angiogenesis of PU scaffolds in vivo. (TIFF 1521 kb)

Additional file 7: **Figure S5.** The effect of platelet-rich plasma (PRP) at different concentrations was evaluated for its effect on rat adipose-derived stem cells (rADSCs) cell viability and expression of angiogenic factor vascular endothelial growth factor (VEGF) and basic fibroblast growth factor (bFGF) in vitro over 14 days. Thee PRP concentrations were evaluated including 2-, 5-, 10- and 15-fold increase that of normal rat blood with a 30-min incubation period. [A] rADSC viability was significantly greater on polyurethane scaffolds with PRP at a concentration 10-fold that of rat blood compared to 2-, 5- and 15-fold over 14 days in culture using alamar blue assay (*p* < 0.05). [B] mRNA expression of VEGF and bFGF by the rADSCs was significantly greater by the rADSCs on the scaffolds treated with PRP at a concentration 10-fold that of rat blood compared to 2-, 5- and 15-fold after 14 days by RT-qPCR. *p* values * < 0.05. (TIFF 1521 kb)

## Abbreviations

ADSC: Adipose-derived stem cells
DMAC: Dimethylacetamide
ECM: Extracellular matrix
PM: Plasma modification
PRP: Platelet-rich plasma
PU: Polyurethane
