# Supplementary material for: Argon plasma surface modification promotes the therapeutic angiogenesis and tissue formation of tissue-engineered scaffolds in vivo by adipose-derived stem cells
Source: Stem Cell Res Ther. 2019 Mar 29;10:110. doi: 10.1186/s13287-019-1195-z (PMC6440049; doi:10.1186/s13287-019-1195-z)
Supplement: Supplementary file 1 — Table S1. Details of flow cytometry antibodies including their fluorescent dye, excitation wavelength and dilution. FITC: fluorescein isothiocyanate, APC: allophycocyanin, PE: phycoerythrin. (DOCX 16 kb) [file 13287_2019_1195_MOESM1_ESM.docx]

|  | CD90 | CD44 | CD34 | CD31 | CD45 |
| --- | --- | --- | --- | --- | --- |
| **Source** | Abcam, Cambridge, UK | | | | |
| **Clone** | HIS51 | OX-50 | ICO-115 | TLD-3A12 | MRC OX-1 |
| **Isotype** | IgG2a | IgG1 | IgG1 | IgG1 | IgG1 |
| **Fluorochrome** | APC | PE | PE | FITC | FITC |
| **Concentration** | 1:1 | 1:1 | 1:1 | 1:1 | 1:1 |
| **Emission/ Excitation wavelength (nm)** | 660/645 | 575/488 | 575/488 | 528/493 | 528/493 |

**Table S1.** **Details of flow cytometry antibodies including their fluorescent dye, excitation wavelength, and dilution.** FITC: fluorescein isothiocyanate, APC: allophycocyanin, PE: phycoerythrin.
