# Supplementary material for: Argon plasma surface modification promotes the therapeutic angiogenesis and tissue formation of tissue-engineered scaffolds in vivo by adipose-derived stem cells
Source: Stem Cell Res Ther. 2019 Mar 29;10:110. doi: 10.1186/s13287-019-1195-z (PMC6440049; doi:10.1186/s13287-019-1195-z)
Supplement: Supplementary file 2 — Table S2. Flow cytometry voltage configurations. FSC: forward scatter, SSC: side scatter, FITC: fluorescein isothiocyanate, PE: phycoerythrin, APC: allophycocyanin. (DOCX 14 kb) [file 13287_2019_1195_MOESM2_ESM.docx]

| Flow Cytometry Configuration | |
| --- | --- |
| **Parameters** | **Voltage** |
| **FSC** | 0 |
| **SSC** | 269 |
| **FITC** | 385 |
| **PE** | 440 |
| **APC** | 610 |

**Table S2.** **Flow cytometry voltage configurations**. FSC: forward scatter, SSC: side scatter, FITC: fluorescein isothiocyanate, PE: phycoerythrin, APC: allophycocyanin.
