# Supplementary figures and images for: Argon plasma surface modification promotes the therapeutic angiogenesis and tissue formation of tissue-engineered scaffolds in vivo by adipose-derived stem cells
Source: Stem Cell Res Ther. 2019 Mar 29;10:110. doi: 10.1186/s13287-019-1195-z (PMC6440049; doi:10.1186/s13287-019-1195-z)

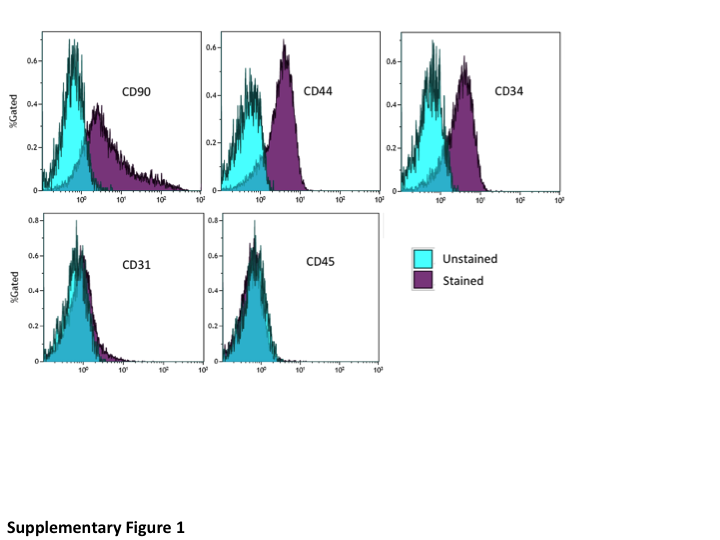

Supplement: Supplementary file 3 — Figure S1. Flow cytometry data of the rat adipose-derived stem cells seeded on the scaffolds. The rat adipose-derived stem cell (rADSCs) stained CD44+/CD34+/CD90+/CD45−/CD31. (TIFF 1521 kb) [file 13287_2019_1195_MOESM3_ESM.tiff]

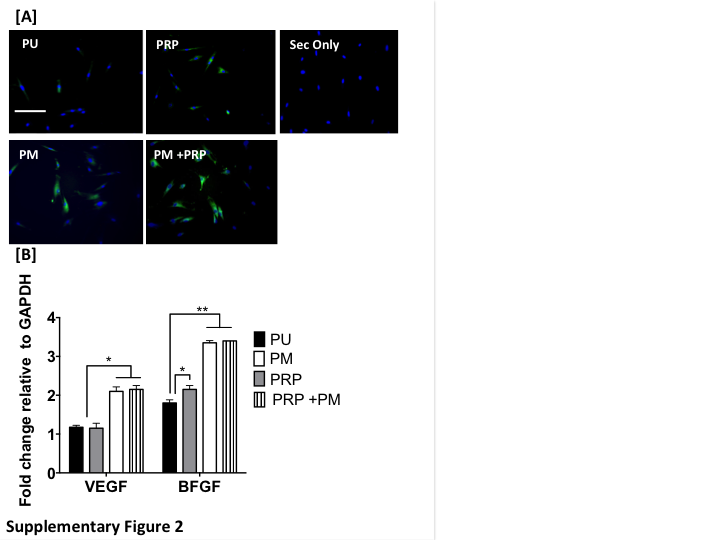

Supplement: Supplementary file 4 — Figure S2. Analysis of angiogenesis using immunocytochemistry and RT-qPCR. [A] Immunocytochemistry of vascular endothelial growth factor (VEGF) from the rat adipose-derived stem cells (rADSCs) after in vitro culture on the modified scaffolds. Note that there was an increased expression of VEGF on PM and PM+PRP scaffolds. Green; VEGF, blue; DAPI. Scale bars 400 μm. [B] RT-qPCR analysis showed VEGF and basic fibroblast growth factor (BFGF) expression of the rADSCs after 14 days of culture. Note the significantly increased levels of expression of VEGF and BFGF on the PM and PRP+PM scaffolds compared to PU and PRP scaffolds (p < 0.05). Fold change is relative to housekeeping gene GAPDH of rADSCs grown on TCP. PU; unmodified scaffolds: PRP; platelet-rich plasma-modified scaffolds, PM; argon-modified scaffold. PRP+PM; platelet-rich plasma and argon modification. Sec; secondary antibody only. p values *< 0.05 and **< 0.01. (TIFF 1521 kb) [file 13287_2019_1195_MOESM4_ESM.tiff]

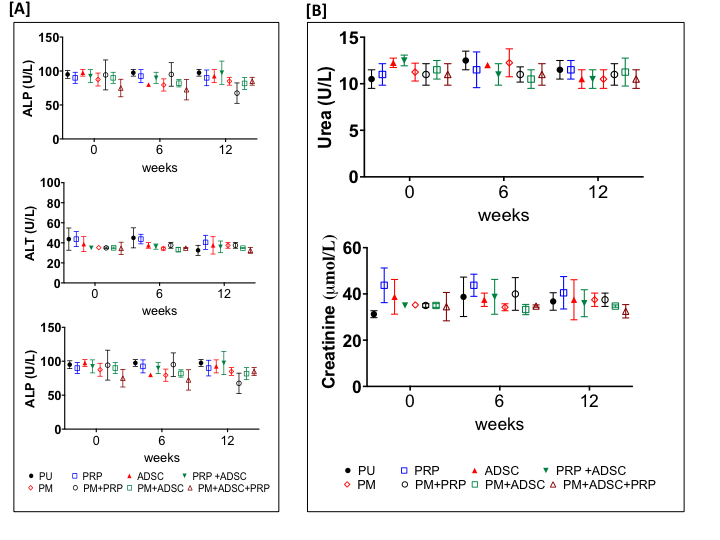

Supplement: Supplementary file 5 — Figure S3. Haematological and biochemistry blood test analysis of the animals over the 12 weeks following implantation of the different scaffolds. [A] Assessment of haematological function. [B] Assessment of liver function. [C] Assessment of renal function. Note no change in haematological, liver function or renal function following implantation of the scaffolds. PU unmodified scaffolds, PRP platelet-rich plasma-modified scaffolds, PM argon-modified scaffold, PRP+PM platelet-rich plasma and argon modification. (ZIP 84 kb) [file 13287_2019_1195_MOESM5_ESM.zip › Slide10.tiff]

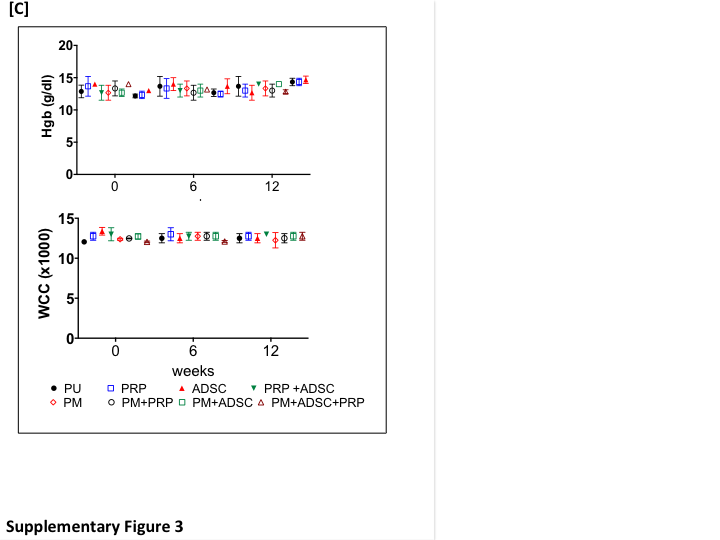

Supplement: Supplementary file 5 — Figure S3. Haematological and biochemistry blood test analysis of the animals over the 12 weeks following implantation of the different scaffolds. [A] Assessment of haematological function. [B] Assessment of liver function. [C] Assessment of renal function. Note no change in haematological, liver function or renal function following implantation of the scaffolds. PU unmodified scaffolds, PRP platelet-rich plasma-modified scaffolds, PM argon-modified scaffold, PRP+PM platelet-rich plasma and argon modification. (ZIP 84 kb) [file 13287_2019_1195_MOESM5_ESM.zip › Slide11.tiff]

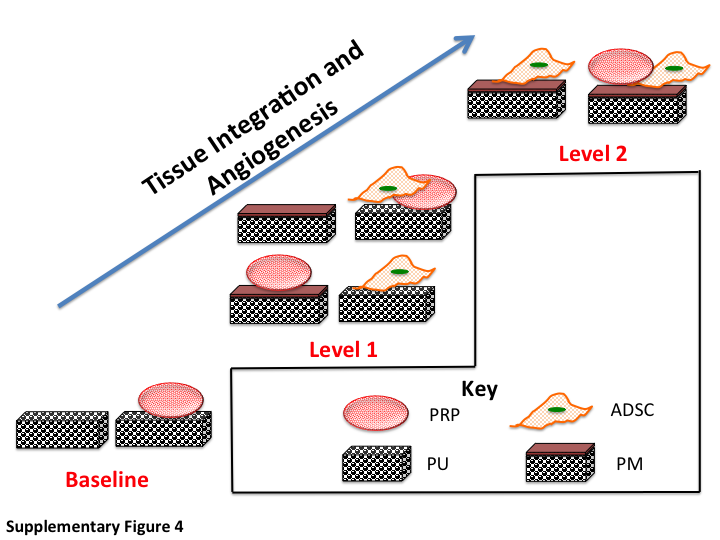

Supplement: Supplementary file 6 — Figure S4. A schematic summary of the effect of PRP and ADSCs on tissue integration and angiogenesis of PU scaffolds in vivo. (TIFF 1521 kb) [file 13287_2019_1195_MOESM6_ESM.tiff]

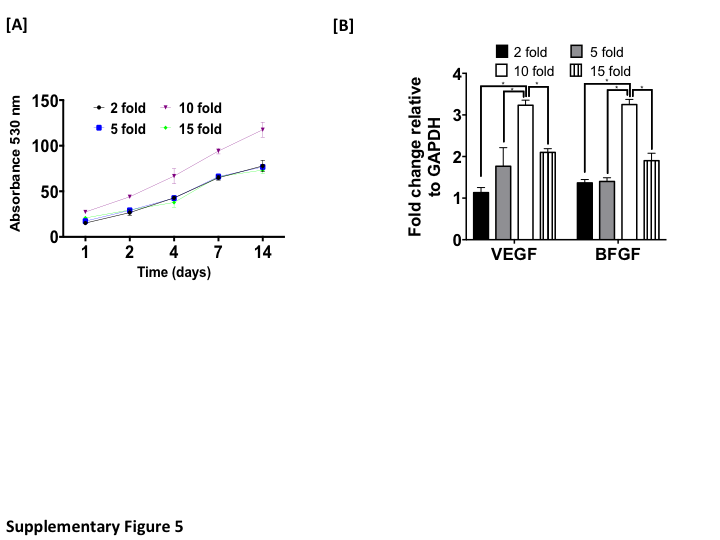

Supplement: Supplementary file 7 — Figure S5. The effect of platelet-rich plasma (PRP) at different concentrations was evaluated for its effect on rat adipose-derived stem cells (rADSCs) cell viability and expression of angiogenic factor vascular endothelial growth factor (VEGF) and basic fibroblast growth factor (bFGF) in vitro over 14 days. Thee PRP concentrations were evaluated including 2-, 5-, 10- and 15-fold increase that of normal rat blood with a 30-min incubation period. [A] rADSC viability was significantly greater on polyurethane scaffolds with PRP at a concentration 10-fold that of rat blood compared to 2-, 5- and 15-fold over 14 days in culture using alamar blue assay (p < 0.05). [B] mRNA expression of VEGF and bFGF by the rADSCs was significantly greater by the rADSCs on the scaffolds treated with PRP at a concentration 10-fold that of rat blood compared to 2-, 5- and 15-fold after 14 days by RT-qPCR. p values * < 0.05. (TIFF 1521 kb) [file 13287_2019_1195_MOESM7_ESM.tiff]
